# Deconcentrating regulation in low- and middle-income country health systems: a proposed ambidextrous solution to problems with professional regulation for doctors and nurses in Kenya and Uganda

**DOI:** 10.1186/s12960-024-00891-3

**Published:** 2024-02-02

**Authors:** Gerry McGivern, Francis Wafula, Gloria Seruwagi, Tina Kiefer, Anita Musiega, Catherine Nakidde, Dosila Ogira, Mike Gill, Mike English

**Affiliations:** 1 King’s College London, London, United Kingdom; 2 Strathmore University, Nairobi, Kenya; 3 Makerere University, Kampala, Uganda; 4 University of Warwick, Coventry, United Kingdom; 5 University College London, London, United Kingdom; 6 University of Oxford, Oxford, United Kingdom

**Keywords:** Regulation, Professional regulation, Medicine, Nursing, Decentralisation, Frontline governance, Decentred regulation, LMICs, Uganda, Kenya

## Abstract

**Background:**

Regulation can improve professional practice and patient care, but is often weakly implemented and enforced in health systems in low- and middle-income countries (LMICs). Taking a de-centred and frontline perspective, we examine national regulatory actors’ and health professionals’ views and experiences of health professional regulation in Kenya and Uganda and discuss how it might be improved in LMICs more generally.

**Methods:**

We conducted large-scale research on professional regulation for doctors and nurses (including midwives) in Uganda and Kenya during 2019–2021. We interviewed 29 national regulatory stakeholders and 47 subnational regulatory actors, doctors, and nurses. We then ran a national survey of Kenyan and Ugandan doctors and nurses, which received 3466 responses. We thematically analysed qualitative data, conducted an exploratory factor analysis of survey data, and validated findings in four focus group discussions.

**Results:**

Kenyan and Ugandan regulators were generally perceived as resource-constrained, remote, and out of touch with health professionals. This resulted in weak regulation that did little to prevent malpractice and inadequate professional education and training. However, interviewees were positive about online licencing and regulation where they had relationships with accessible regulators. Building on these positive findings, we propose an ambidextrous approach to improving regulation in LMIC health systems, which we term *deconcentrating regulation*. This involves developing online licencing and streamlining regulatory administration to make efficiency savings, freeing regulatory resources. These resources should then be used to develop connected subnational regulatory offices, enhance relations between regulators and health professionals, and address problems at local level.

**Conclusion:**

Professional regulation for doctors and nurses in Kenya and Uganda is generally perceived as weak. Yet these professionals are more positive about online licencing and regulation where they have relationships with regulators. Building on these positive findings, we propose deconcentrating regulation as a solution to regulatory problems in LMICs. However, we note resource, cultural and political barriers to its effective implementation.

**Supplementary Information:**

The online version contains supplementary material available at 10.1186/s12960-024-00891-3.

## Background

Health workers play a central role in health systems. Having sufficient numbers of well trained and motivated health workers is essential to achieving universal health coverage and meeting Sustainable Development Goals [1, 2]. However, there is a human-resources for health crises in many LMICs. There are too few health workers [1, 3]. Pay is often poor and irregular. Many health workers are demotivated [4–8]. Strikes [4, 6, 9], absenteeism [4, 6–8, 10, 11], nepotism and malpractice are widespread [10, 12].

We examine how regulation of health workers may contribute towards or mitigate these problems. Regulation can enhance health workers’ professional practice [13, 14]. However, it is often weakly implemented and enforced in LMIC health systems. This is due to reasons including regulators having limited resources, regulatory capture, and corruption [15–19].

Strengthening health systems requires more health professionals to be trained, but this must be balanced against maintaining and regulating standards of professional practice and training [20]. Yet little research has examined the way health professionals are regulated in LMICs, although a few systemic policy overviews suggest it is problematic in many African [21], South and East Asian [8, 18, 20, 22, 23] countries. There are also related concerns about poor regulation and standards of health professional education and training in LMICs, particularly in private universities and training colleges [15, 21, 24–26], including in Africa [3, 21, 25, 27], India [22, 23, 25, 28], Thailand [25], Cambodia and Vietnam [29].

Very few *empirical* studies have examined health professional regulation in LMICs. One recent empirical study in Ethiopia found regulation of health workers to be weak [17]. Another describes India nursing regulators as under-resourced, disengaged from professional representatives, with limited capacity to address malpractice, improve practice or training, and professional practice ‘completely ignored’ [30].

The lack of empirical research on health professional regulation is problematic, as regulation often fails due to lack of ‘contextual fit’ between regulation on ‘paper’ and the lived circumstances in which this is enacted [31]. We therefore need to better understand the practical norms, perceptions, experiences, and relationships affecting health professional regulation and practices on the ‘frontline’. By ‘frontline’ regulation we mean regulatory practices that are directly implemented, applied, enforced, and affect behaviours in the setting where regulators and those they regulate work [32–39].

Moreover, while government ministries and statutory regulators are responsible for regulating health professionals, professional associations develop codes of professional conduct and ethics that regulatory standards are based on and influence professional behaviour and practice. Health professional regulation and regulatory outcomes are therefore co-produced between governments, regulators and professionals at national and local levels [15, 40]. Accordingly, we also consider regulation from a ‘decentred’ perspective [41], examining how a range of related views, activities, social, cultural, political and practical norms, central and local stakeholders affect regulatory relationships, behaviours, and outcomes [8, 15, 36, 42–45].

A decentred regulatory focus also raises questions about the appropriate balance between regulatory centralisation and decentralisation. In many LMICs, including Kenya and Uganda, regulation is overseen by regulators and government ministries centrally. However, governance of health systems has been devolved and decentralised. *Decentralisation* aims to bring governance ‘close to ground’ and motivate local participation in improvement [46]. However, in practice, these aims are often undermined by politics, bureaucracy, limited resources, and inadequate technical skill at local level [46–48], so decentralisation has delivered ‘mixed results’ overall [46, 48, 49]. An alternative to decentralisation is *deconcentration* [46, 47], involving subnational governmental offices connected to common central structures providing skills, resources and accountability nationally. However, we need to better understand what aspects of centralisation and decentralisation [46] are beneficial to regulation in particular contexts.

## Methods

### Data collection

We conducted large-scale mixed-methods research on professional regulation for nurses (including midwives) and doctors in Kenya and Uganda in 2019–21, in urban/central and rural/remote settings. We purposefully sampled these two professions, LMICs and geographies to explore variations and generalisability in our findings, and how variations in regulatory approaches and contexts affected them. Sampling choices were also practically limited by our research project’s resource and time constraints.

We first conducted qualitative semi-structured interviews with 29 national-level Kenyan and Ugandan regulatory stakeholders (October 2019–February 2020). These interviewees were recruited to represent the range of groups and organisations involved in and affected by medical and nursing regulation. We then interviewed 47 doctors, nurses (including midwives), and other subnational regulatory actors in two Ugandan districts (June 2020) and two Kenyan counties (September 2020). We sampled and conducted interviewees until a point of data saturation, where we understood the views and experiences of the professionals and regulatory stakeholders in these local areas. We asked common open interview questions about professional regulation, enabling comparison across professional and national groups.

Next, we ran an online/paper-based survey (April–June 2021) open to all doctors, nurses (and midwives), medical and nursing interns and students in Kenya and Uganda. We opted for a convenience sample, aiming to reach as many participants as possible. We therefore publicised and distributed the online survey via email, social media, and professional associations. We also distributed paper copies of the survey questionnaire to doctors and nurses in rural counties/districts, where internet access was limited, to ensure responses from rural areas. Our survey collected 3466 responses.

The survey explored views and experiences of professional regulation too. It drew on measures validated in previous survey-based research on professional regulation [45, 50] and new questions testing the generalisability of key themes emerging in interviews. Survey participants responded to statements (e.g., ‘Regulation has a positive effect on my professional practice’) on the Likert scale (1 = strongly disagree to 5 = strongly agree) and demographic questions (e.g., about country, profession, age), showing variations in responses by characteristics (see details in Table 1).

**Table 1:** Survey respondents

Country	Kenya	Uganda	Total
Doctors	259	340	599
Nurses/midwives	704	1268	1972
Medical and nurse interns	108	265	373
Medical and nursing students	182	340	522
Total	1253	2213	3466

Finally, we conducted four online/in-person focus group discussions (September 2021) to validate emerging findings and explanations with Kenyan and Ugandan doctors, nurses, and regulators. These contained separate groups of Ugandan and Kenyan nurses and doctors (involving 33 participants in total). Focus groups provided further qualitative data (see Table 2).

**Table 2:** Interviewees

Country	Kenya	Uganda
National level stakeholder interviews (N = 29; conducted Oct 2019 to Feb 2020)	Ministry of Health (× 2) Kenya Health Professions Oversight Authority Nursing Council of Kenya Kenya Medical Association Kenya Medical Practitioners, Pharmacists & Dentists’ Union (× 2) National Nurses Association of Kenya Kenyan National Union of Nurses University of Nairobi, College of Health Science Kenya Medical Training College Kenya Healthcare Federation Total N = 12	Ugandan Ministry of Health (× 2) Ugandan Ministry of Education (× 2) President’s Health Monitoring Unit Uganda Medical & Dental Practitioners Council Uganda Medical Association Uganda Nurses & Midwives Council Uganda Nurses & Midwives Union (× 3) National Health Consumers Association Uganda Healthcare Federation Uganda Allied Health Professions Council Pharmaceutical Society of Uganda Makerere School of Medicine Public Health Nurses College Total N = 17
County/ district-level interviews (N = 47)	12 in County C 7 in County D 10 doctors 8 nurses 1 County representative Total N = 19 (September 2020)	16 in District A 12 in District B 7 doctors 21 nurses Total N = 28 (June 2020)
FGDs (N = 33; Sept 2021)	6 Kenyan nurses (online) 6 Kenyan doctors (online) Total N = 12	9 Uganda nurses (online & face-to-face) 12 Ugandan doctors (online) Total N = 21
Total participants	N = 43	N = 66
	N = 109	

### Data analysis

Interviews and focus group discussions were digitally recorded and transcribed. We thematically analysed [51] qualitative interview and focus group data. We developed thematic codes drawing on literature and empirical data. We identified key themes that were prevalent in data (e.g., ‘weak regulation’) and/or empirically and theoretically significant (e.g., positive views of ‘relational and local regulation’). We combined sub-themes (e.g., ‘relational & local regulation’ and ‘calls for regulatory decentralisation’) into aggregate themes (e.g., ‘improving regulation through deconcentration’). We present thematic codes and illustrative data extracts in Table 3.

**Table 3:** Interview extracts illustrating regulatory themes

Aggregate theme	Empirical sub-themes	Illustrative thematic qualitative extract
Weak regulation	Inadequate resources to regulate	“[KMPDC] has been challenged in terms of their scope of work. The Council of less than 20 people with no branches within the country, [KMPDC] are bombed with so much, including training of medical students.” (Kenya Medical Association representative) “UMDPC is poorly funded, employs a few people, who are usually overwhelmed with work.” (Ugandan Doctor, FGD) “We had challenges, we were understaffed, didn’t have enough resources.” (Uganda Nursing & Midwifery Council Representative) “Key challenges? We are a small team; the country is big everybody thinks you are the only people who can provide solutions.” (Health Monitoring Unit representative, Uganda)
	Remote regulators	[KMPDC] don’t have any presence in the counties, so there was that gap. They don’t have a presence on the ground. You cannot supervise counties from Nairobi. In five years, not a single person from the board has ever visited. They [regulators] are not in touch with what is happening on the ground.” (Doctor 7, Kenyan county C) [Regulators] “not really in touch with the nursing fraternity on the ground.” (Nurse 18, Kenyan county V) “For us, who are upcountry [far from the Ugandan capital city], we haven’t had any direct engagement with [regulators].” (Doctor 4, Ugandan district B) “I’ve never seen them [UNMC] in more than ten years. Those people are comfortable in those buildings [Kampala offices] but we are here suffering, nurses not complying, professionalism is dying because those guys are not coming out of their offices.” (Nurse 2, Ugandan district B)
	Regulators’ more focused on collecting licencing fees than regulating professional practice	“The only time I have interacted with KMPDC was when I was getting the practising license and then when I am getting my annual retention license. You don’t pay, that is the only thing they look at. Coming to the ground to supervise the facilities, in my entire practice, I’ve never seen them.” (Doctor 5, Kenyan country T) “The only time you interface with the registration body is when they need the [license] fee. If a patient complains of malpractice, nobody is interested. Regulatory bodies are not functional, not looking at professionalism or quality of service but they are more interested in collecting revenue.” (Doctor 3, Ugandan, district) “As long as the money is going to the Nursing Council of Kenya, your interaction is very nice. But if they don’t need money from you, we don’t see them.” (Kenyan nurse, FGD) “[Ugandan nurses] don’t have any guidance, we only hear, ‘you have to pay.’” (Nurse 2, Ugandan district B)
	Inadequate resources to address health system failures blamed on individual professionals	“The regulator is toothless when it comes to funding classrooms or hospitals. It is a bigger thing than the regulator.” (Kenyan Training provider representative) “It is difficult to differentiate between a system failure and a professional failure. Doctors and nurses are being accused unfairly by the Kenyan public and sometimes by the Kenyan media because the system is so weak, and it does not support them.” (Kenyan Nursing Council representative) “The whole system is dysfunctional. The public forgets. The doctor gets all the blame on behalf of the system.” (Kenya Medical Association representative) “If you want to blame, on face value it looks like the doctor’s or nurse’s medical malpractice or professional negligence but there is always an institutional gap.” (Doctor, Kenyan county) “If something goes wrong in a hospital, the blame always will be on that nurse but conditions on the ground, that’s what really kills.” (Uganda Nurses & Midwives Council representative)
	Weakly enforced regulation	“Basically, they [regulations] don’t exist in practice.” (National stakeholder, Uganda) “There’s a general perception that measures are not deterrent and punitive enough. But those punitive actions, should really come last after restorative measures. That’s something that the public doesn’t understand.” (Regulatory representative, Uganda) “No regulatory body, nobody monitors. There is no consequence for doing wrong. So, most of us are driven by the oath and the institution of medical training tends to train you to care, look after patients and do the right thing but even if you don’t do the right thing, the consequences are not there really. If you have a weak regulatory body, you can get away with a lot.” (Uganda doctor 3): “Regulators are lenient. There are no punitive measures. That encourages impunity. So, they must be able to bite, to deter others from being negligent in future.” (Kenyan training provider representative)
	Weak social accountability	“Nobody honestly cares about the common mwanainchi [citizen]. There is no level of accountability.” (Kenyan Medical Association representative) “Our people are a bit cautious, most of them don’t complain. A complaint usually comes from a backlash from the community.” (Doctor 10, Kenyan county V) “We need to do a lot of health promotion. They [patients] even fear to ask a doctor whether he [sic] is qualified.” (Ugandan Ministry of Health official) “Some of the patients are manipulated because they don’t know their rights.” (Doctor 10, Uganda district B)
Inadequate regulation of health professional education & training	Inadequate regulation of medical & nursing education	“We are producing professional health workers who are half-baked. None has any experience, training hands-on that you need to see a patient. Stop opening schools every county, training masses, producing lower quality professionals. So, it’s a matter of regulation. Medical training will one day just dilute, anybody will be a doctor.” (Doctor 12, Kenyan county V) “Private medical schools, it’s a mess. The quality of our doctors is down, they’re not properly trained. I don’t see any regulatory mechanism for who gets into medical school, who qualifies, there’s no unified standards. Everybody qualifies doctors in any way they like.” (Ugandan doctor 3) “These [regulatory] bodies are not visible anywhere, they should be visiting [medical] schools mostly to engage with the finalists, so that when you qualify, you know to come to this body, and this will be required of you.” (Doctor 7, Ugandan district A) “As professional councils, the manpower we have is limited to assessing whether training providers have standards. We’re unable to get into whether teachers can teach competently.” (Regulator, Uganda) “Trainees are half-baked because of these mushrooming institutions. If you get money, you can start a nursing school. This thing is affecting nurses. Everywhere there is a problem. The training period is limited, and their practical period is too short. They don’t care. A patient is not a big deal to them because they are after money.” (Nurse 2, Ugandan district B) “Training needs to be improved aggressively monitored. Not to just leave it to the institution” (Doctor 25, Uganda district A)
	Overenrolling students	“If you look at education, the ratio of a tutor [to students] is 1:60 in government [training institutions], in private [training institutions] it is 1:200 but 1:10 in a class, that’s the recommendation of WHO. We shall produce fake substandard people.” (Uganda Nurses & Midwives Union representative) “[X] university; they are admitting 400 medical students to train in a place where they are only allowed to admit a maximum of 150. So, this is likely to compromise the quality of training. And I was telling them I am worried that even the products [health professionals] you are going to give us is not going to meet the standard that we have.” (Kenyan regulator 3) “Someone just opens the school with the aim of getting money with 1,000 students. Where do you get the patients from to practice on? You can expect fake nurses.” (Nurse 9, Ugandan district B)
	Inadequate internships & mentoring	“The regulator has failed in ensuring proper training and mentorship of the younger doctors. Many internship centres don’t have equipment, enough lecturers. Doctors who graduate from these universities don’t have all the required skills and knowledge.” (Medical organisation representative, Kenya)
Streamlining online regulatory administration	Developing online (re)licencing	“We don’t have to travel, it’s very easy. Once I’ve done my CPD, I renew my license, click, and pay with M-Pesa and you print your certificate. Before we used to travel to Nairobi. That would take a week. Now it’s a few minutes.” (Doctor 7, Kenyan county T) “The Nursing Council of Kenya are doing well, even the registration or renewal of licenses is online.” (Nurse, Kenyan county) “We register online. Tt doesn’t even take 10 min. Before, we used to involve in transport.” (Doctor, 10 Ugandan district B) “I am going to renew [my nursing license online]. It has been fast; registration it is fast now.” (Nurse 7, Ugandan district A)
	Streamlining regulatory administration	“Time and effort are focused on registration, licensing and collecting fees, which is a huge job that someone else could do, so regulators are free to regulate the profession. Currently enforcement is geared towards people who have not paid licences, instead of practice, ethics, and conduct.” (Ministry of Health official, Uganda)
Improving regulation through deconcentration	Relational & local regulation more effective	“It used to be difficult. Once registration was brought to this hospital, there is now no problem. When there are problems they [regional office] forward them to the Council, then they come, see problems, and get solutions.” (Nurse, Ugandan district A) “[The Uganda Health Monitoring Unit] are doing a good job, at least they come… monitor… you feel like they have guided… Sometimes, instead of guiding or finding out, they are rude and want to arrest you, but at least we have interfaced with them. Other regulators, no.” (Nurse 10, Ugandan district B) “The Nursing Council seems to be more vibrant than the Medical Council. The perception is that nurses would be more regulated. I think it’s just the system setup; the person doing the day-to-day running of the hospital is the nurse. Nurses seem to have a direction.” (Kenyan medical FGD)
	Calls for regulatory decentralisation to local level	“Decentralise to the Counties and strengthen and empower those at the County level to oversee regulation. In every county have a regulator and officers in touch with what is happening to the professionals down here.” (Doctor 10, Kenyan county V) “The council needs also to decentralise the operations, to have these regional offices. It should be easier for us to go to these regional offices to assess our issues.” (Doctor 7, Ugandan district B) “We embrace the need for us to expand this office outside Kampala.” (Uganda medical regulatory representative)

After presenting key themes in qualitative interview data, we also show percentages of respondents agreeing with individual survey questions that illustrate these themes’ wider generalisability. These illustrative individual survey results are independent of the factor analysis we conducted, as discussed below.

To analyse quantitative survey data, we used an exploratory factor analysis (Direct Oblim; based on Eigenvalues greater than 1, accepting items with factor loadings over 0.50 and intercorrelations of less than 0.30) [52]. We established thematic factors, which survey questions were aggregated into. We present factors related to key themes discussed in qualitative findings. Additional file 1: Appendix 1 lists questions associated with each factor. Appendices 2 and 3 show means, standard deviations, correlations, and reliability for factors. Appendices 4 to 7 show mean responses for these factors by professional group, which we use to show the generalisability and variations by respondents in relation to key themes.

## Results

Below we present key themes in our empirical data. Additional file 1: Appendix 8 provides information about the Kenyan and Ugandan medical and nursing professions, and their regulation and training, which contextualise these themes.

### Weak regulation and its underlying causes

Regulation in both the Kenyan and Ugandan health systems was generally perceived as ‘*weak’*. Kenyan and Ugandan regulators were seen to have inadequate resources and staff, to be *‘remote’*, *‘out of touch’* with clinical practice ‘*on the ground’*, and more focused on collecting licencing fees than regulating professional practice. For example, a representative from the Uganda Nurses and Midwives Council (UNMC), which regulates over 50,000 practising nurses, noted that UNMC had just 23 staff, and only two on the government payroll, so *‘depend so much on revenue we get from registration licensure.’* In our survey, 33% agreed ‘I have had sufficient contact with staff from my regulator in the last year’. 43% agreed ‘my regulator is just interested in collecting registration and licence fees’.

Interviewees described a public perception that health system regulation *‘doesn’t exist in practice’* (Ugandan national patient representative) and is *‘weakly’* enforced by *‘lenient’* regulators, which ‘*encourages impunity’* (Kenyan training provider). However, representatives of both professional associations and regulators commented that individual professionals were often blamed for problems resulting from wider health system failures. Regulators investigating malpractice therefore described taking ‘*restorative measures’* before issuing sanctions.

Social accountability provides a potential mechanism for challenging professional malpractice. However, interviews described weak social accountability, with public and patients who are *‘cautious, don’t complain’*, *‘ignorant of their rights’* (Doctor, Kenyan county), with little knowledge about how to challenge malpractice. Regulators were seen to be unlikely to investigate malpractice until there was a health professional scandal in the media. Policymakers and regulators in Kenya and Uganda likewise acknowledged the need for publicity informing the public of health care rights.

In our survey, 45% of respondents had ‘witnessed medical or nursing malpractice’. 65% reported having ‘had concerns about a professional colleague’s ability to do their job’, of which only 7% ‘reported the concerning colleague to their professional regulator’. 41% agreed ‘my regulator does not deal effectively with malpractice’. Qualitative responses in our survey suggested that, where addressed, concerns about health professionals tended to be dealt with informally.

However, despite its problems, most interviewees believed they understood regulatory standards and that regulation had a positive overall impact. This was due to the possibility of regulatory sanctions motivating health professionals to be *‘more careful’* (Doctor 15, Ugandan district)*.* In our survey, 77% were ‘familiar with my regulator’s standards’. 81% agreed ‘Regulation has a positive effect on my professional practice.’

Additional file 1: Appendices 4–7 shows the aggregated mean responses by professional groups for four factors entitled: *Perceptions of regulatory effectiveness* (Additional file 1: Appendix 4); *Witnessing malpractice and negligence* (Additional file 1: Appendix 5); *Regulatory effectiveness in dealing with malpractice* (Additional file 1: Appendix 6); and *understanding of regulatory standards* (Additional file 1: Appendix 7)*.* Appendices 4 and 6 show that Kenyan nurses perceived the highest and Kenyan doctors the lowest regulatory effectiveness of the four main professional groups. Interns and students were more positive about regulatory effectiveness than fully qualified professionals. Additional file 1: Appendix 5 shows that Kenya doctors reported witnessing the most malpractice and negligence. Doctors were more likely than nurses to report witnessing malpractice and negligence. Interns more likely to do so than students. Additional file 1: Appendix 7 shows that Kenyan nurses were most and Ugandan nurses least likely to report understanding regulatory standards. These appendices also show that the themes we describe can be generalised across professions and countries.

### Inadequate regulation of health professional education and training

Interviewees also described weak regulation and declining standards of training, internships, supervision, and mentoring of health professionals. This was seen to be undermining professionalism, skills, and knowledge. For example, Nurse 19 (Ugandan district) described *‘mushrooming training schools’* producing *‘half-baked trainees’* putting ‘*the nursing profession in a shambles’.* Interviewees expressed particular concern about new private universities and training colleges over-enrolling students, up to ‘*ten times above the limit’* (Kenyan regulatory representative). In our survey, 38% agreed ‘newly qualified members of my profession lack the skills they need to provide high quality patient care in this country’.

Some regulatory representatives acknowledge that regulation of health professional training was inadequate but said that this too was due to lack of regulatory staff and resources. In Uganda, interviewees also suggested that poor health professional training/education was due to the Ministry of Education and Sports (rather than the Ministry of Health) being responsible for health professionals’ education/training, and not understanding skills health professionals need.

### Streamlining online regulatory administration

In contrast with general views of regulation, health professionals were positive about the development of online (re)licencing. Although difficult in remote areas with limited internet, online (re)licencing was generally seen to reduce the time, cost and effort involved in renewing licences, particularly for health professionals in remote areas. Doctor 7 (Kenyan County) noted: *‘It’s very easy. I renew my license, click, and pay with M-PESA [mobile phone-based money transfer]. Before we used to travel to Nairobi. That would take a week. Now it’s a few minutes.’*

At the time of interviews, online licencing had just been established for Kenyan doctors and nurses, Ugandan doctors, and was under development for Uganda nurses. A joint online licensing portal for Ugandan doctors, nurses, and allied health professionals (https://www.ehealthlicense.go.ug) has since been launched. A Ugandan regulatory representative suggested that streamlining regulatory administration, centralising registration and licensing across regulators could produce efficiency savings, allowing regulators to focus on monitoring health professionals’ practice, conduct and ethics.

### Positive views of local relational regulation

Interviewees were also positive about regulation where they had relationships with accessible local regulators. As noted above, the Nursing Council of Kenya (NCK) was perceived as the most effective regulator in our survey (see Factor in Additional file 1: Appendix 4: perceptions of regulatory effectiveness). Similarly, more Kenyan nurse interviewees described having good relationships with their professional regulator than in other professional groups. For example, Nurse 14 (Kenyan county V) commented, *‘[NCK] really assist us, support us. Our working relationship with the regulator has been very cordial. They make time and visit us.’*

In one Ugandan district we researched, there is a UNMC office based in a regional referral hospital. This office is responsible for managing local nursing licence renewals and records, disciplinary cases and regulating local training colleges. Contrasting with general perceptions of UNMC, Ugandan nurses described this office as accessible and effective. A nurse (central Ugandan district) commented: *‘Once registration was brought to this hospital, there’s now no problem. [UNMC] see problems and get solutions.’*

The Health Monitoring Unit (HMU) is a regulator monitoring health services, professional training, and investigating malpractice across Uganda. Ugandan interviewees reported that the HMU was generally *‘feared’* for arresting health workers accused of malpractice. However, some doctors and nurses were nonetheless positive about HMU because it engaged with health professionals. Ugandan doctor 10, who worked in the remote Ugandan district where regulation was generally viewed poorly, noted: *‘[HMU] are doing a good job. At least they come, monitor, you feel like they have guided… They are rude and want to arrest you, but at least we have interfaced with them. Others [regulators], no.’*

Some interviewees called for regulatory ‘decentralisation’, so regulators could develop relationships with professionals and address problems affecting the ‘frontline’. For example, when asked what would most improve health professional regulation, Kenyan Doctor 10, who worked in the remote Kenyan county, commented: *‘Decentralise… have a regulator in touch with what’s happening to the professionals down here.’*

However, Kenyan interviewees also warned of *‘political interference’* in local health systems, and ‘*politically connected’* individuals who *‘bend the rules, know the right thing but cannot do it because they think their office will be taken away’* (Doctor 3, Kenyan County V). Therefore, there is a risk of decentralised regulation being undermined by local level politics.

### Summary of themes

Table 3 summarises and presents qualitative data illustrating the four aggregate themes and sub-themes discussed above: (1) *Weak regulation* (including *Inadequate resources to regulate; remote regulators; regulators more focused on collecting licencing fees than regulating professional practice; inadequate resources to address health system failures blamed on individual professionals; weakly enforced regulation; weak social accountability*); (2) *Inadequate regulation of health professional education and training* (including *over-enrolling students; inadequate internships and mentoring*); (3) *Streamlining online regulatory administration* (including *developing online (re)licencing; streamlining regulatory administration*); and (4) *Improving regulation through deconcentration* (including *relational and local regulation more effective; calls for regulatory decentralisation to local level*).

## Discussion

Our interview and survey data suggest that Kenyan and Ugandan health professional regulators are inadequately resourced, generally seen to be remote, out of touch, and more focused on collecting licence fees than regulating professional practice, education, and training. Consequently, malpractice may be common but rarely reported or sanctioned by regulators. These findings echo previous research on regulation of health professionals [15–19, 21–23, 30] and professional training and education [15, 21–29].

However, we also present novel research findings about where health professionals viewed regulation positively. First, online (re)licencing was seen as quick, easy, and effective. Second, health professionals were more positive about regulation where they had relationships with accessible regulators. These novel findings reflect ‘responsive’ [44] and ‘relational’ regulation theory [32, 43], which hypothesise that good regulatory relationships enhance understanding of how and why people should comply, so increase compliance, and improve regulators’ understanding of compliance levels and how to improve regulation [32, 43–45]. Accordingly, some doctors in remote areas called for regulatory decentralisation, so that regulators were brought closer to ‘frontline’ professionals and problems. Building on these two positive findings, we propose a novel ambidextrous approach to improving regulation in LMICs.

### Deconcentrating regulation, streamlining online regulatory administration, and barriers to improvement

We propose *deconcentrating* [46, 47] regulation by developing subnational regulatory offices connected to a common national structure. This is distinct from *decentralisation,* which involves independent governance at subnational level [46, 47]. Our findings raise concerns about decentralised independent regulators lacking resources, expertise and undermining common national standards of professional regulation and practice. Deconcentrating regulation would also bring regulators closer to professional practice, enabling them to better detect and address problems, but while sharing national resources, expertise, information, learning and standards. Common problems in subnational areas could then be addressed by improving national regulatory standards, training, or guidance in ways reflecting learning in responsive regulation theory [44].

Deconcentrated regulation could also contribute towards creating cohesive policy ecosystems at subnational level, where different regulators work closely with other decentralised institutional mechanisms (e.g., internal security, social protection, and education). Working systemically could prevent problems falling between the responsibilities of different organisations too, and thus remaining unaddressed.

However, there are barriers to developing deconcentrated regulation. First and foremost, most LMIC health systems are severely resource-constrained, which directly and indirectly undermines health professionals’ practice. Deconcentrating regulation would require new resources, although local regulatory offices could use existing public resources (e.g., rooms in local healthcare facilities), so would not be costly. But where might new resources come from? Echoing a Ugandan regulator, we suggest that developing online (re)licensing and streamlining centralised regulatory administration, and perhaps sharing administration across regulators as is happening in Uganda, can produce efficiency savings releasing resources for developing deconcentrated regulation.

**Fig. 1:**
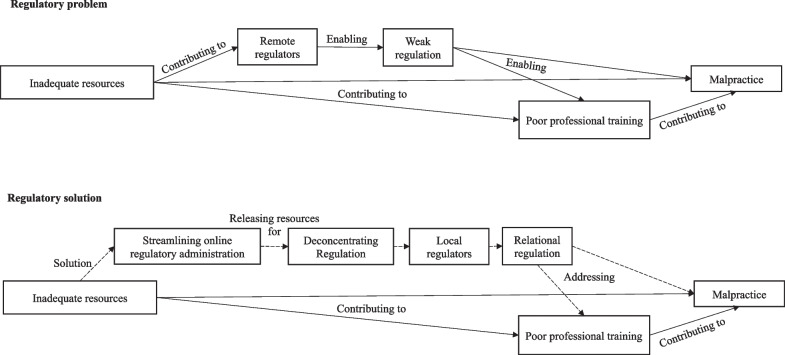
Model of regulatory problem and solution of deconcentrated regulation

Figure 1 summarises the regulatory problems we found and our proposed solution. We show inadequate resources contributing towards poor professional practice and training, remote and weak regulation, and malpractice. Our solution involves streamlining online regulatory administration, releasing resources for deconcentrating regulation, developing local regulatory offices and regulatory relationships, mitigating poor professional practice and training.

Our proposals chime with a recent literature review on decentralisation in LMICs, which argues that health systems are most efficient when combining centralisation, providing economies of scale, with decentralisation of functions requiring ‘close to ground’ decisions [46]. Our proposals also reflect ‘the trick’ in designing ‘high reliability’ organisation (e.g., nuclear power stations), which is ‘simultaneous centralisation and decentralisation’ [53]. Decentralisation enables people to independently interpret what is going on, improvise and prevent errors in complex settings but depends on centralised standards, learning from previous experiences, structures for addressing errors, and enculturing people into appropriate norms [53].

Yet a second barrier is cultural. Implementing deconcentrated regulation requires new regulatory ‘hardware’ (resources, structures, and technologies) and ‘software’ (cultures, values, relationships, and trust) [54–56]. Our findings show weak regulation and standards of health professional education, training and practice reinforcing one another. Developing professional cultures focused on improving practice is ‘hard’ in LMICs, due to limited staff, training, and resources [14]. However, there are examples of well lead and resourced health care organisations developing successful improvement cultures in LMICs [57] and ‘friendly and supportive’ inspection of health facilities increasing compliance [56]. Yet resources do need to be devoted to developing cultural ‘software’ for deconcentrated health system regulation.

A final barrier is politics. LMIC health systems are often undermined by politics and nepotism [4, 6, 10]. Regulation specifically may be used politically to deflect blame for wider systemic failures [39, 58]. Indeed, interviewees described health professionals being blamed for failings in wider health systems, which does little to address problems. Decentralising and deconcentrating regulation, moving monitoring closer to the ‘front line’ of professional practice, can enhance professional engagement, relationships, enable improvement ‘close to ground’ and integration of subnational ecosystems but also risk local professional and political capture [4, 16, 33, 35, 38, 55]. Yet by developing national-local regulatory accountability relationships and transparency mechanisms [46], deconcentrating regulation is less likely to be undermined by politics.

### Research limitations and opportunities for future research

We acknowledge our paper’s limitations, which provide opportunities for future research. Our research was conducted on two professions in just two East African Countries, so more work is needed to test the generalisability of our findings and proposals in LMICs. Research exploring whether health professionals in LMICs are generally positive about online licencing and relational local regulation would be particularly useful. More research is also needed on regulation of health professional education and training in LMICs, which our study and other recent research [15, 21–29] suggests is undermining health professional practice.

Finally, we lack empirical evidence about whether and how deconcentrated regulation does improve professional practice, behaviour, and patient care as we suggest. The Ugandan Allied Health Professionals Council recently introduced online licencing and established ten regional regulatory offices (https://www.ahpc.ug/region.php), which reflects the model of deconcentrated regulation we propose, so would provide an interesting test case. However, more empirical research on deconcentrated regulation in a wider range of LMICs health systems is needed.

## Conclusion

We conducted empirical research on professional regulation for doctors and nurses in Kenya and Uganda. We found regulators generally perceived as lacking resources, ‘remote’, ‘out of touch’ with ‘frontline’ professionals and failing to address malpractice or inadequate training standards for health professionals. However, doctors and nurses in Uganda and Kenya were positive about online licencing and regulation where they have relationships with regulators. Building on these findings, we propose an ambidextrous approach to improving health professional regulation in LMICs, involving streamlining regulatory administration and online relicensing, releasing resources for deconcentrating, and supporting regulation at subnational level.

### Supplementary Information

**Additional file 1. **Supplementary Appendices.

## Data Availability

Datasets generated and analysed during the study are not publicly available, to protect research participant’s privacy, but available from the corresponding author on reasonable request.
