# Supplementary material for: Deconcentrating regulation in low- and middle-income country health systems: a proposed ambidextrous solution to problems with professional regulation for doctors and nurses in Kenya and Uganda
Source: Hum Resour Health. 2024 Feb 2;22:13. doi: 10.1186/s12960-024-00891-3 (PMC10835984; doi:10.1186/s12960-024-00891-3)
Supplement: Supplementary file 1 — Additional file 1. Supplementary Appendices. [file 12960_2024_891_MOESM1_ESM.docx]

**Appendix 1: Questions forming factors**

| ***Figure 1: Factor - Perception of regulatory effectiveness*** *(correlation 0.822)*  My regulator makes sure that its members actually meet the standards in practice  My regulator’s disciplinary procedures are effective  I believe that my regulator’s standards reflect good practice  I am confident that my regulator regulates my profession well  The regulations that govern my profession are appropriate  My regulator communicates well with members of my profession |
| --- |
| ***Figure 2: Factor - Understanding of regulatory standards*** *(correlation 0.662)*  I know what the standards actually require me to do  I have a clear sense of whether or not I am complying with my regulator’s standards  I am familiar with my regulator’s standards |
| ***Figure 3: Factor - Witnessing malpractice and negligence*** *(Correlation 0.802)*  I have witnessed medical or nursing malpractice where I work  I have witnessed medical or nursing negligence where I work |
| ***Figure 4: Factor - Regulatory efficacy dealing with malpractice*** *(Correlation 0.621)*  My regulator is effective in preventing ‘quacks’ from practicing  The sanctions my regulator can impose deter malpractice  I understand my regulator’s process for handling complaints made against professionals |

**Appendix 2: Means and standard deviations for factors by group**

|  | Perception of regulatory effectiveness | | Understanding of regulatory standards | | Witnessing malpractice & negligence | | Regulatory effectiveness dealing with malpractice | |
| --- | --- | --- | --- | --- | --- | --- | --- | --- |
|  | *Mean* | *SD* | *Mean* | *SD* | *Mean* | *SD* | *Mean* | *SD* |
| All | 3.59 | 0.77 | 3.84 | 0.77 | 3.07 | 1.11 | 3.29 | 0.85 |
| Kenya Doctor | 3.35 | 0.74 | 3.87 | 0.68 | 3.45 | 0.92 | 2.92 | 0.91 |
| Uganda Doctor | 3.5 | 0.82 | 3.9 | 0.83 | 3.36 | 1.01 | 3.06 | 0.93 |
| Kenya nurse | 3.63 | 0.74 | 4.03 | 0.79 | 3.18 | 1.04 | 3.36 | 0.83 |
| Uganda nurse | 3.55 | 0.82 | 3.75 | 0.79 | 2.88 | 1.18 | 3.29 | 0.86 |
| Kenya med & nurse intern | 3.75 | 0.6 | 3.93 | 0.68 | 3.3 | 0.82 | 3.55 | 0.7 |
| Uganda med & nurse intern | 3.49 | 0.73 | 3.78 | 0.69 | 3.27 | 1.09 | 3.19 | 0.79 |
| Kenya med & nurse student | 3.92 | 0.63 | 3.92 | 0.79 | 2.91 | 1.07 | 3.58 | 0.82 |
| Uganda med & nurse student | 3.77 | 0.67 | 3.71 | 0.68 | 2.8 | 1.1 | 3.47 | 0.67 |

**Appendix 3: Means, standard deviations and correlations of four main measures**

|  | Mean | Standard Deviation | 1 | 2 | 3 | 4 |
| --- | --- | --- | --- | --- | --- | --- |
| 1. Perception of regulatory effectiveness | 3.59 | 0.77 | (.82) |  |  |  |
| 2. Understanding of regulatory standards | 3.84 | 0.77 | .35^**^ | (.66) |  |  |
| 3. Witnessing malpractice & negligence | 3.07 | 1.11 | -.22 | -.02 | (.80) |  |
| 4. Regulatory effectiveness dealing with malpractice | 3.29 | 0.85 | .49^**^ | .20^**^ | -.15 | (.62) |

Note. ** p > .01 (two-tailed). Cronbach Alpha in brackets in diagonal.

**Appendix 8: Regulatory bodies and professional training in Kenya and Uganda**

In Kenya, there are 9,132 medical practitioners licenced to practice^[[1]](#footnote-1)^ and 58,247 registered nurses/midwives in 2019^[[2]](#footnote-2)^. The ratio of doctors and nurses to population in Kenya falls well below World Health Organization recommendations (1, 2). The Kenyan health system has gone through major changes following devolution to county-level responsibility in 2013. It has also faced major challenges, including industrial action by healthcare professionals and perceptions of widespread clinical malpractice. These have negatively affected health care quality, safety, and professional engagement (2-4).

*The Kenya Medical Practitioners and Dentists Council* (KMPDC) regulates doctors and medical education and training, and The *Nursing Council of Kenya* (NCK) regulates Kenyan nurses, midwives, and nursing/midwifery training in Kenya. Kenyan doctors and nurses/midwives must register with their regulator and renew their professional licenses annually by paying fees and demonstrating sufficient continuing professional development (CPD). The Kenyan *Ministry of Health* is responsible for health care and health professionals’ education/training in the country. *The* *Kenya Health Professionals Oversight Authority (KHPOA;* established in 2017) provides oversight of Kenyan health practice and all its healthcare regulatory bodies. Eight public and three private universities are approved to provide medical training in Kenya^[[3]](#footnote-3)^, 22 universities and 80 mid-level colleges to train nurses at certificate, diploma, and degree-levels^[[4]](#footnote-4)^ . Graduate-level doctors and nurses undergo a yearlong internship before being licensed.

In Uganda, there are 7,742 medical and dental practitioners, although just 4,417 renewed their annual practicing licenses in 2021^[[5]](#footnote-5)^. There are 81,435 registered nurses and midwives in Uganda but only 52,335 have active practicing licenses^[[6]](#footnote-6)^. This equates to 1.238 nurses per 1000 population (2018 figures)^[[7]](#footnote-7)^. These data suggests that doctors and nurses may be leaving the Ugandan health system for high income countries, retiring from practice, or practising without a renewed licence. The ratio of doctors and nurses to population in Uganda also already falls well below World Health Organization recommendations (5). The Ugandan health system faces other acute resource limitations and rising levels of reported clinical negligence and related claims (6, 7).

*The Uganda Medical and Dental Practitioners Council* (UMDPC) regulates doctors and medical education and trainingand *The* *Uganda Nursing and Midwives Council* (UNMC) regulates nurses/midwives and their education and training in Uganda. Medical licences are renewed annually, and nursing/midwifery licences every three years. The *Ministry of Health* is responsible for Ugandan health care, while the *Ministry of Education and Sports* is responsible for all education and training (including for health professionals). *The* *Health Monitoring Unit* (established in 2009; reporting to State House and/or the Ugandan President), monitors performance and delivery of health services, professional training, and investigates criminality and malpractice across Uganda. In Uganda, 11 universities (six private) are licenced to train doctors to degree level^[[8]](#footnote-8)^ and 72 nursing colleges and 15 universities (many private) are licences to provide nursing/midwifery training at certificate, diploma, and degree-level.^[[9]](#footnote-9)^ Degree-level Ugandan doctors and nurses/midwives also undergo a one-year internship before being licenced.

**References**

1. Kinuthia R, Verani A, Gross J, Kiriinya R, Hepburn K, Kioko J, et al. The development of task sharing policy and guidelines in Kenya. Human Resources for Health. 2022;20(1):1-12.

2. Koon AD. When doctors strike: making sense of professional organizing in Kenya. Journal of health politics, policy and law. 2021;46(4):653-76.

3. WHO. Primary Health Care Systems (PRIMASYS): Case Study from Kenya Geneva: World Health Organisation; 2017.

4. Waithaka D, Kagwanja N, Nzinga J, Tsofa B, Leli H, Mataza C, et al. Prolonged health worker strikes in Kenya-perspectives and experiences of frontline health managers and local communities in Kilifi County. International journal for equity in health. 2020;19:1-15.

5. Ajari EE, Ojilong D. Assessment of the preparedness of the Ugandan health care system to tackle more COVID-19 cases. Journal of Global Health. 2020;10(2).

6. WHO. Primary Health Care Systems (PRIMSYS): Case study from Uganda. Geneva: World Health Organization; 2017.

7. Nassaka F. Medical Negligence. The Indepenent 2015.

1. <http://kmpdc.go.ke/Registers/Dashboard/Dashboard.php> [↑](#footnote-ref-1)
2. <https://www.statista.com/statistics/1240331/number-of-nurses-in-kenya-by-category/> [↑](#footnote-ref-2)
3. https://kmpdc.go.ke/undergraduate-training/ [↑](#footnote-ref-3)
4. https://nckenya.com/approved-training-institutions/ [↑](#footnote-ref-4)
5. <https://umdpc.com/Resources/Brochures/Brochure%202021.pdf> [↑](#footnote-ref-5)
6. [https://unmc.ug](https://unmc.ug/) [↑](#footnote-ref-6)
7. <https://data.worldbank.org/indicator/SH.MED.NUMW.P3?locations=UG-KE> [↑](#footnote-ref-7)
8. <https://umdpc.com/resources.php> [↑](#footnote-ref-8)
9. <https://unmc.ug/recognized-schools/> [↑](#footnote-ref-9)
